# Analysis of codon usage patterns in complete plastomes of four medicinal *Polygonatum* species (Asparagaceae)

**DOI:** 10.3389/fgene.2024.1401013

**Published:** 2024-09-19

**Authors:** Naixing Shi, Yiwen Yuan, Renjie Huang, Guosong Wen

**Affiliations:** College of Agronomy and Biotechnology, Yunnan Agricultural University, Kunming, China

**Keywords:** *Polygonatum* species, chloroplast genome, codon usage bias, mutation pressure, natural selection, evolution

## Abstract

Polygonati Rhizoma and Polygonati odorati Rhizoma, known as “Huangjing” and “Yuzhu” in China, are medicinal *Polygonatum* species resources with top-grade medical and edible properties. The chloroplast (cp) genome has been used to study species diversity, evolution, and breeding of species for applications in genetic engineering. Codon usage bias (CUB), a common and complex natural phenomenon, is essential for studies of codon optimization of exogenous genes, genetic engineering, and molecular evolution. However, the CUB of medicinal *Polygonatum* species chloroplast genomes has not been systematically studied. In our study, a detailed analysis of CUB was performed in the medicinal *Polygonatum* species chloroplast genomes. We investigated the codon bias of 204 plastid protein-coding genes (PCGs) in 4 medicinal *Polygonatum* species using CodonW and CUSP online software. Through the analysis of the codon bias index, we found that the medicinal *Polygonatum* species chloroplast genomes had weak codon usage bias. In addition, our results also showed a high preference for AT bases in medicinal *Polygonatum* species chloroplast genomes, and the preference to use AT-ending codons was observed in these species chloroplast genomes. The neutrality plot, ENC plot, PR2-Bias plot, and correspondence analysis showed that compared with mutation pressure, natural selection was the most important factor of CUB. Based on the comparative analysis of high-frequency codons and high expression codons, we also determined the 10-11 optimal codons of investigative medicinal *Polygonatum* species. Furthermore, the result of RSCU-based cluster analysis showed that the genetic relationship between different medicinal *Polygonatum* species could be well reflected. This study provided an essential understanding of CUB and evolution in the medicinal *Polygonatum* species chloroplast genomes.

## 1 Introduction

The *Polygonatum* Mill. comprises approximately 80 species and is species-rich genus in Polygonateae, which occur in temperate Northern Hemisphere regions (Wang J et al., 2022; Wang et al., 2023; Floden and Schilling, 2018). Many species of this genus are significant medicinal plants in China, and have been widely used as Traditional Chinese Medicines (TCM) to treat fatigue, weakness, diabetes, cough, and loss of appetite for thousands of years (Zhao et al., 2018; Chen et al., 2021). The Chinese Pharmacopoeia embodied four species (*Polygonatum odoratum*, *Polygonatum sibiricum*, *Polygonatum cyrtonema*, *Polygonatum kingianum*) as medicinal *Polygonatum* species (Chinese Pharmacopoeia Commission, 2020). In addition, as medicinal and edible plants, most of this genus greatly benefits human health, which is often used as one of the main raw materials of health food (Cui et al., 2018; Bai et al., 2021; Xiao et al., 2023). Because of the remarkable medicinal and edible value of medicinal *Polygonatum* species, hundreds of drugs and functional foods, such as medicine, food, and drink whose raw materials are based on *Polygonati Rhizoma* and *Polygonati odorati Rhizoma*, have been developed and applied in China (https://db.yaozh.com/zhongyaocai) (Guo et al., 2022).

As important organelles in green plants, chloroplasts play a crucial role in photosynthesis. According to their functions, there are four categories of chloroplast genes: self-replication genes, photosynthesis genes, biosynthesis-related genes, and unknown function genes (Cao et al., 2022; Sugiura, 1995). The chloroplast genome has the characteristics of relatively small size, uniparental inheritance, multicopy numbers, and highly conserved genomic structure (Daniell et al., 2016). These features are of great advantage in genetic transformation (James and Daniell, 2005; Wang et al., 2009). Therefore, it has attracted extensive attention from relevant researchers in recent years (Kumar and Ling, 2021; Singhal et al., 2023; Basso et al., 2020). To date, chloroplast genome engineering has been used in many biotechnology applications (Daniell et al., 2016). With the rapid development of high-throughput sequencing technologies, the number of chloroplast genomes has been increased using next-generation sequencing (NGS) technology in the NCBI (https://www.ncbi.nlm.nih.gov/) and NGDC (https://ngdc.cncb.ac.cn/?lang = zh) databases. The chloroplast genomes of four medicinal *Polygonatum* species (*P. odoratum*, *P. sibiricum*, *P. cyrtonema*, and *P. kingianum*) have also attracted significant attention and have been reported by many researchers (Wang et al., 2023; Xia et al., 2022; Floden and Schilling, 2018).

The frequency of synonymous codons probability differs in all genes and organisms due to the comprehensive effects of mutation, selection, and genetic drift (Parvathy et al., 2021; Salim and Cavalcanti, 2008). Organisms and genes tend to encode amino acids using one or more specific synonymous codons called codon usage bias (CUB). Studying the CUB of organisms has important biological implications. Because closely related species have similar patterns of codon bias, therefore, CUB analysis can reveal species evolutionary, horizontal gene transfer and so on (Athey et al., 2017; Tuller, 2011). Besides, studies have found that CUB is vital for a multitude of cellular processes, for example, influencing the stability and efficiency of transcription, as well as the structure, expression, function of proteins (Chakraborty et al., 2020). Hence analyzing CUB can be utilized to optimize expression vectors in genetic engineering, thereby enhancing the expression levels of target genes (Kwon et al., 2016). As a universal natural phenomenon, it exists widely in different organisms (Wang et al., 2011). At present, numerous studies on CUB of medical plant have been reported, e.g., *Dendrobium* species (Wang X. S et al., 2022), *Lilium* species (Dai et al., 2024), *Aconitum* species (Yang et al., 2023). However, CUB in medicinal *Polygonatum* species has not been exhaustively investigated. The quality of Polygonati Rhizoma and Polygonati odorati Rhizoma products is critical to the pharmaceutical and food industries. By studying the CUB of medicinal *Polygonatum* species cp genomes, stable genetic transformation can be more efficiently constructed and increase the expression level of the target gene in genetic transformation research. Moreover, it will also provide an important research basis for revealing the genetic relationships and evolutionary analysis of these medicinal *Polygonatum* species.

## 2 Materials and methods

### 2.1 Sequence data

**FIGURE 1:**
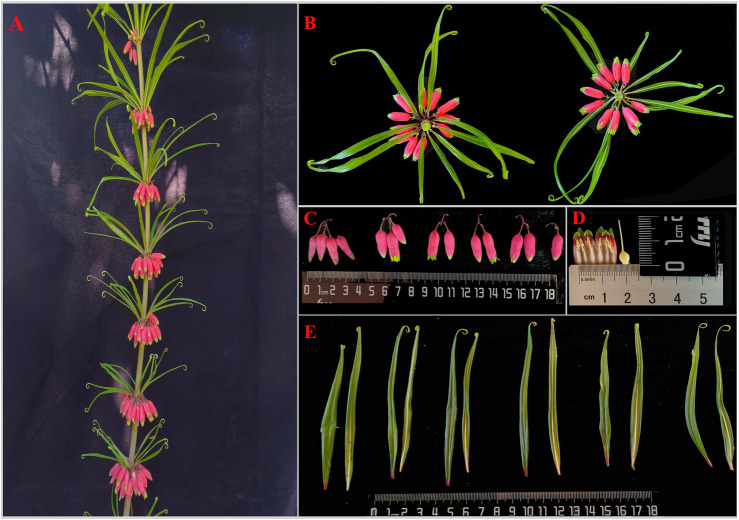
*Polygonatum kingianum* Coll. et Hemsl. **(A)** individual, **(B)** whorled leaf, **(C)** flower and umbel, **(D)** anatomy of flower, **(E)** leaves.

**FIGURE 2:**
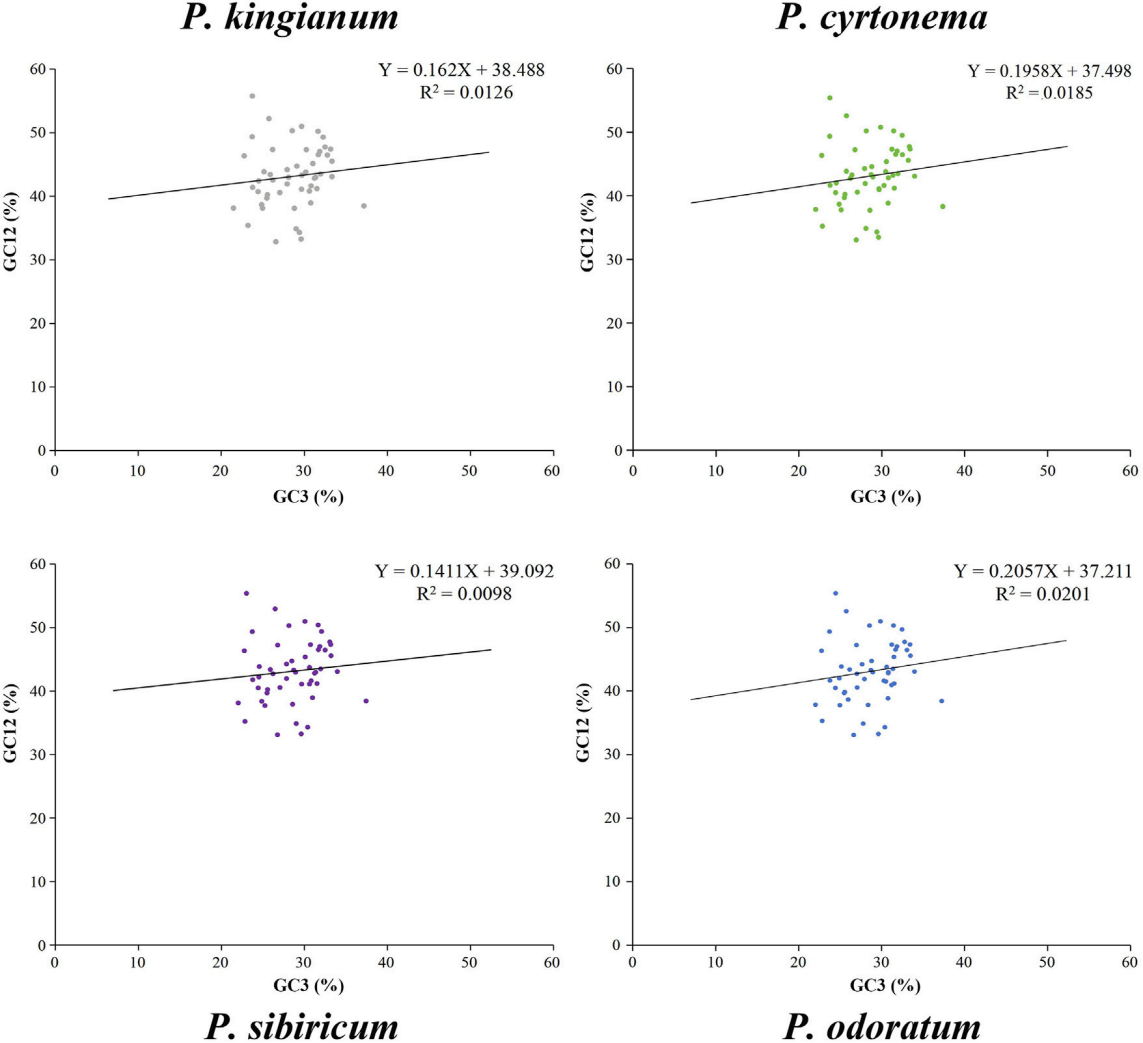
Plastome map of *Polygonatum kingianum*. Genes inside and outside the circle were transcribed clockwise and counter-clockwise, respectively. Cp genome structure:LSC, large single copy region; SSC, small single copy region; IR, inverted repeat. The genes belonging to different functional groups are different color-coded. In the inner circle, the lighter gray indicates the AT content, and the darker gray indicates the GC content.

The voucher specimen (SNX3) was collected from the Yuxin TCM plantation base (N25.76104 and E103.75594) and deposited at Yunnan Agricultural University, Kunming, China (Figure 1). Leaf tissues from SNX3 were dried with silica gel. The total DNA was extracted from 100 mg of dried leaves using the modified CTAB method (Porebski et al., 1997). Then, a paired-end (PE) library of 150 bp was constructed using the total DNA and performed on the Illumina Hiseq 2500 sequencing platform. The PE reads were trimmed for adapter and low-quality reads (Phred score <30) using NGS QC Toolkit v.2.3.3 software (Patel and Jain, 2012). The *P. kingianum* cp genome was assembled using GetOrganelle v.1.6.4 (Jin et al., 2020). After assembly, the circular cp genomes were annotated using online tools CpGAVAS2 (Shi et al., 2019) and GeSeq (Tillich et al., 2017) based on the reference cp genome (GenBank: MZ286313). The annotated cp genome sequence was submitted to the GenBank database of the NCBI (Accession: PP315902). The plastome map (Figure 2) was drawn by OrganellarGenomeDRAW (OGDRAW) online (Lohse et al., 2007). In addition, we also downloaded the other medicinal *Polygonatum* species chloroplast genomes from the NGDC database (https://ngdc.cncb.ac.cn/?lang = zh), including two species (*P. cyrtonema*, GenBank number: ON872700; *P. sibiricum*, GenBank number: OQ532972) previously reported by us and one species (*P. odoratum*, GenBank number: MZ150858) reported by other scholars. Information on medicinal *Polygonatum* species is shown in Table 1.

**TABLE 1:** Detailed information of four chloroplast genomes of medicinal Polygonatum species.

Species	GenBank number	Total sequence	CDSs number (before fltering)	CDSs number (after fltering)	Total sequence (after fltering)	L_aa (after fltering)
*P. kingianum*	PP315902	1,55,714	85	51	62,736	20,912
*P. cyrtonema*	ON872700	1,55,521	85	51	62,703	20,901
*P. sibiricum*	OQ532972	1,55,513	86	51	62,703	20,901
*P. odoratum*	MZ150858	1,54,577	85	51	62,652	20,884

### 2.2 Sequence processing

In order to reduce sampling errors and redundancy of the CUB analysis, all protein-coding sequences (CDSs) from chloroplast genomes were selected for subsequent analysis based on the following principles: 1) excluding duplicate genes, 2) CDSs with length greater than 300 bp were retained, 3) retaining CDSs without intermediate stop codons and erroneous bases. 4) CDSs starting with a start codon (ATG) and ending with a stop codon (TAG, TGA, TAA) were retained (Wang et al., 2020; Li et al., 2016).

### 2.3 Analysis of codon usage index

All the codon usage indicators were calculated through CodonW 1.4.2 software (Peden, 1999) and the CUSP program of EMBOSS (Rice et al., 2000), including (1) effective number of codon (ENC), (2) relative synonymous codon usage (RSCU), (3) codon number (L_aa), (4) overall GC content of genes and the GC content at the first, second and third base position of codons (GC1, GC2, GC3), (5) the G or C content of the third base of a synonymous codon (GC3s), (6) The frequency of base A, T, G, and C at the third position of codons (A3_S_, T3_S_, G3_S_, C3_S_).

### 2.4 Neutrality plot analysis

A neutrality plot was conducted using GC12 as the ordinate and GC3 as the abscissa to evaluate the degree of influences from mutation pressure and natural selection. GC12 represents the average GC content of GC1 and GC2. If there is a significant correlation between GC12 and GC3, it indicates that codon usage is mainly affected by mutation pressure. In contrast, if there is no correlation between GC12 and GC3, it means that natural selection is the main driving force in influencing codon usage bias (Sueoka, 1988).

### 2.5 ENC-plot analysis

ENC-plot analysis is a routine analysis to explore whether the codon preference of a specific gene is caused by mutation or selection pressure. The ENC-plot is used to draw a two-dimensional scatter plot with GC3 as the abscissa and ENC value as the ordinate. The expected curve was calculated based on the following formula: ENC_exp_ = 2+GC3_S_+29/[GC3_S_^2^+(1-GC3_S_)^2^]. If the corresponding points are distant from the expected curve, this reveals that the gene will be influenced by natural selection. Mutational effects may be the dominant factor if the points fall near the standard curve (Wright, 1990).

We also analyzed the ENC frequency ratio distribution to determine further the main factors affecting the CUB. The value of the ENC ratio was calculated according to the formula: ENC_ratio_ = (ENC_exp_-ENC_obs_)/ENC_exp_.

### 2.6 Parity Rule 2 plot analysis

Parity rule 2 (PR2) plot analysis is one of the methods to examine the effects of mutation pressure and natural selection on codon bias. PR2 plot, as a graphical analysis, is widely used to reveal the relationship between the variation of the four bases at the third codon position and the codon usage bias (Sueoka, 1999). In our study, the graphic were established with A3_S_/(A3_S_ + T3_S_) and G3_S_/(G3_S_ + C3_S_) as the x- and y-axis, respectively. In the PR2 plot, the center point means that the codon usage of genes is only affected by mutational pressure (Galtier and Lobry, 1997). The vector emitted from the center point represents the degree and direction of the codon usage bias (Li et al., 2023).

### 2.7 Statistical analysis

Correspondence analysis (COA) is a commonly multivariate statistical approach to analyze codon usage patterns (James and McCulloch, 1990). We performed a correlation analysis based on the parameters (GC1, GC2, GC3, GCall, ENC, and L_aa) obtained by the CodonW 1.4.4. SPSS 20.0 software was used to COA based on Spearman’s rank correlation method.

### 2.8 Determination of optimal codons

The codon with RSCU>1 is considered as the high-frequency codon (Yu et al., 2012). Furthermore, all tested genes were arranged from large to small according to the ENC values. The 10% of the genes were screened from the top and bottom to establish high-and low-expression gene groups, respectively (Gao et al., 2024). The RSCU values of the two datasets were then calculated. Codons with ΔRSCU ≥0.08 (ΔRSCU = RSCU_high-expression_-RSCU_low-expression_) were defined as high-expression codons (Liu et al., 2020). Finally, the codon meeting both high frequency and high expression was identified as the optimal codon (Liu and Xue, 2004) for the chloroplast genome of medicinal *Polygonatum* species.

### 2.9 RSCU-based cluster analysis

We explored the relationship of the four medicinal *Polygonatum* species using a hierarchical clustering method. The OriginPro 2019b (OriginLab Corporation, 2019) was employed to construct the RSCU value matrix, and calculate the average distance of these species.

## 3 Results

### 3.1 Nucleotide composition analysis

**FIGURE 3:**
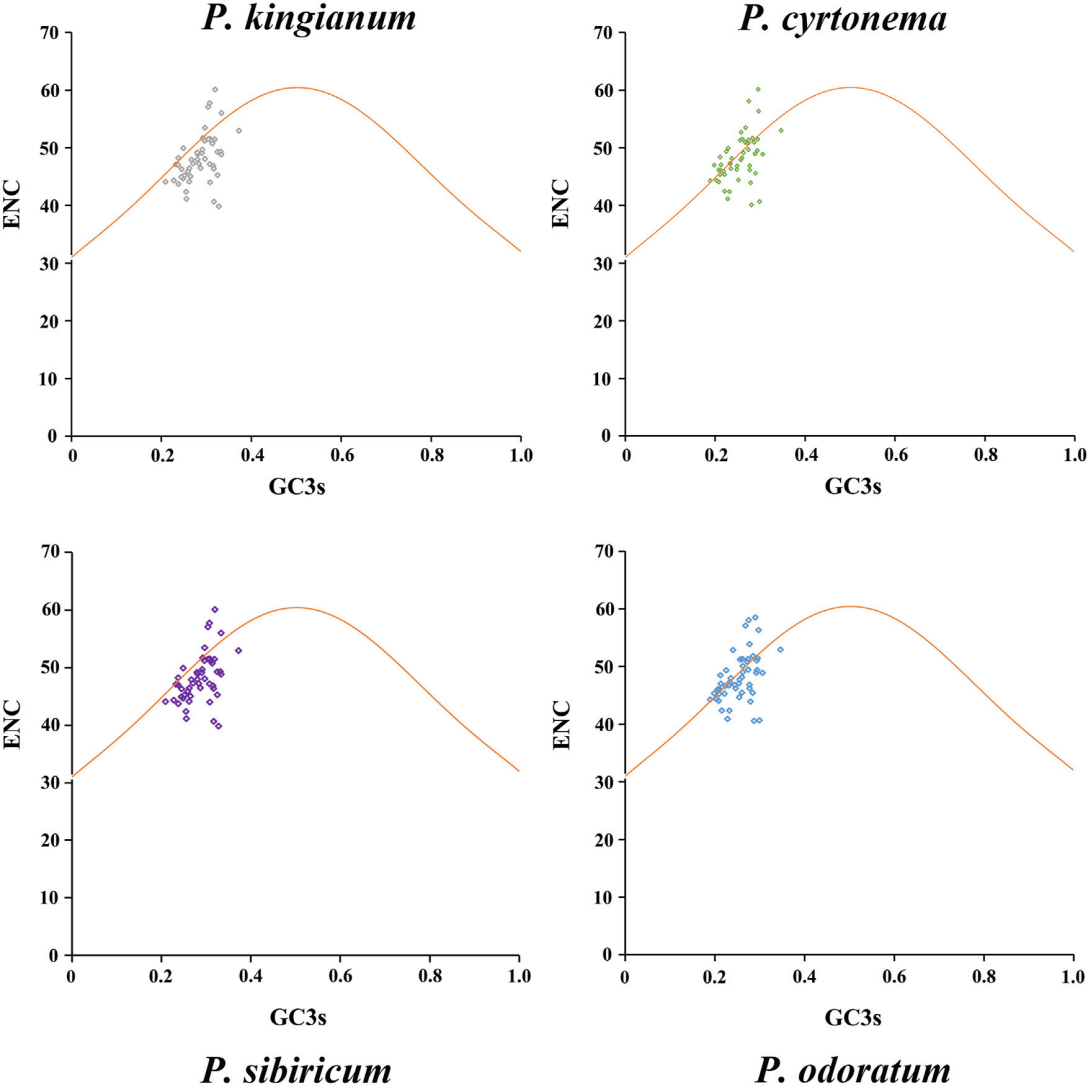
The related GC content of tested cp genes in medicinal *Polygonatum* species. The color variation is closely related to GC, GC1, GC2, and GC3 content. Green-to-red color indicates low to high percentage (%).

**FIGURE 4:**
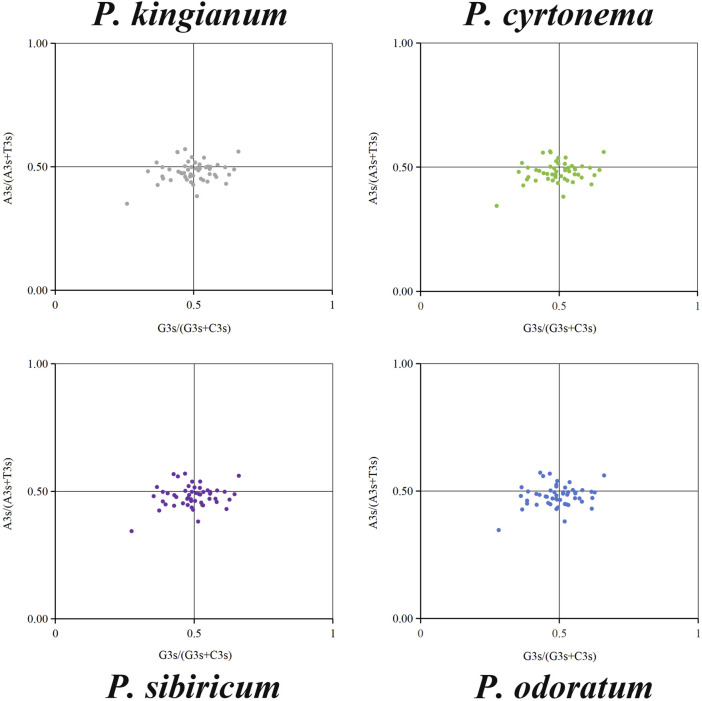
RSCU values for 21 amino acids (64 codons) in all tested cp genes of medicinal *Polygonatum* species. The different colors on the bar graph correspond to the color of the codon below the *X*-axis.

The 204 CDSs were screened from four medicinal *Polygonatum* species for subsequent analysis (Table 2). These tested CDSs mainly included photosynthesis genes, ribosome genes, self-replication genes, hypothetical chloroplast reading frames, and other genes. All codon usage indexes (ENC, RSCU, L_aa, GCall, GC1, GC2, GC3, GC3s, A3_S_, T3_S_, G3_S_, C3_S_) were calculated as shown in Supplementary Tables S3, S4. The third codon positions (GC1, GC2, and GC3) for each CDS in the four medicinal *Polygonatum* species exhibited a similar trend of GC content, with GC1 > GC2 > GC3 (Figure 3). It is considered that when ENC≤35, the gene has significant codon bias and *vice versa* (Comeron and Aguade, 1998). The ENC values of the four medicinal *Polygonatum* species had a weak preference for codons (40.1–60.11 for *P. sibiricum*, 40.58–58.53 for *P.odoratum*, 40.1–60.11 for *P.cyrtonema*, and 39.83–60.11 for *P. kingianum*, respectively). The average ENC values for self-replication, photosynthesis, unknown function, and other genes ranged from 47.33 to 47.42, 47.42 to 47.66, 49.38 to 49.72, and 53.86 to 54.74. Among the above four categories of genes, *psbA* gene has the strongest codon usage preference (ENC values: 39.83–40.58), while *ycf3* gene has the weakest codon bias, (ENC values: 58.53.83–60.11), as shown in Supplementary Table S3. RSCU is calculated as the ratio of the expected frequency of amino acid synonymous codon usage to its observed frequency (Shields et al., 1988). We calculated the RSCU values of each amino acid in the four medicinal *Polygonatum* species (Supplementary Table S5; Figure 4). There were 30 codons with an RSCU greater than 1.00, which were defined as high-frequency codons. Among these high-frequency codons, 29 of these ended with A/T, including UUU, UUA, CUU, AUU, GUU, GUA, UCU, UCA, AGU, CCU, CCA, ACU, ACA, GCU, GCA, UAU, UAA, CAU, CAA, AAU, AAA, GAU, GAA, UGU, CGU, CGA, AGA, GGU, GGA, while only one ended with G (UUG). The codon UUA (Leu, RSCU = 1.92–1.93) had the highest RSCU value in these high-frequency codons.

**TABLE 2:** List of screened genes in the chloroplast genomes of medicinal Polygonatum species.

Category of Genes	Group of gene	Name of gene
Self-replication	Ribosomal protein (small subunit)	*rps2, rps3, rps4, rps7c, rps8, rps11, rps14, rps18*
	Ribosomal protein (large subunit)	*rpl14, rpl16, rpl20, rpl22*
	RNA polymerase	*rpoA, rpoB, rpoC1, rpoC2*
Genes for photosynthesis	Subunits of photosystem I	*psaA, psaB*
	Subunits of photosystem II	*psbA, psbB, psbC, psbD*
	Subunits of cytochrome	*petA, petB, petD*
	Subunits of ATP synthase	*atpA, atpB, atpE, atpF* ^a^ *, atpI*
	Large subunit of Rubisco	*rbcL*
	Subunits of NADH dehydrogenase	*ndhA, ndhB* ^a^ ^,^ ^c^ *, ndhC, ndhD, ndhE, ndhF ndhG, ndhH, ndhI, ndhJ, ndhK*
Other genes	Maturase	*matK*
	Envelope membrane protein	*cemA*
	Subunit of acetyl-CoA	*accD*
	Synthesis gene	*ccsA*
	ATP-dependent protease	*clpP*
	Component of TIC complex	*ycf1* ^d^
Genes of unknown function	Conserved open reading frames	*ycf2* ^c^ *, ycf3* ^b^ *, ycf4*

^a^ Gene containing one intron.

^b^ Gene containing two introns.

^c^ Duplicated genes.

^d^ Partial duplicated gene.

### 3.2 Neutrality plot analysis

**FIGURE 5:**
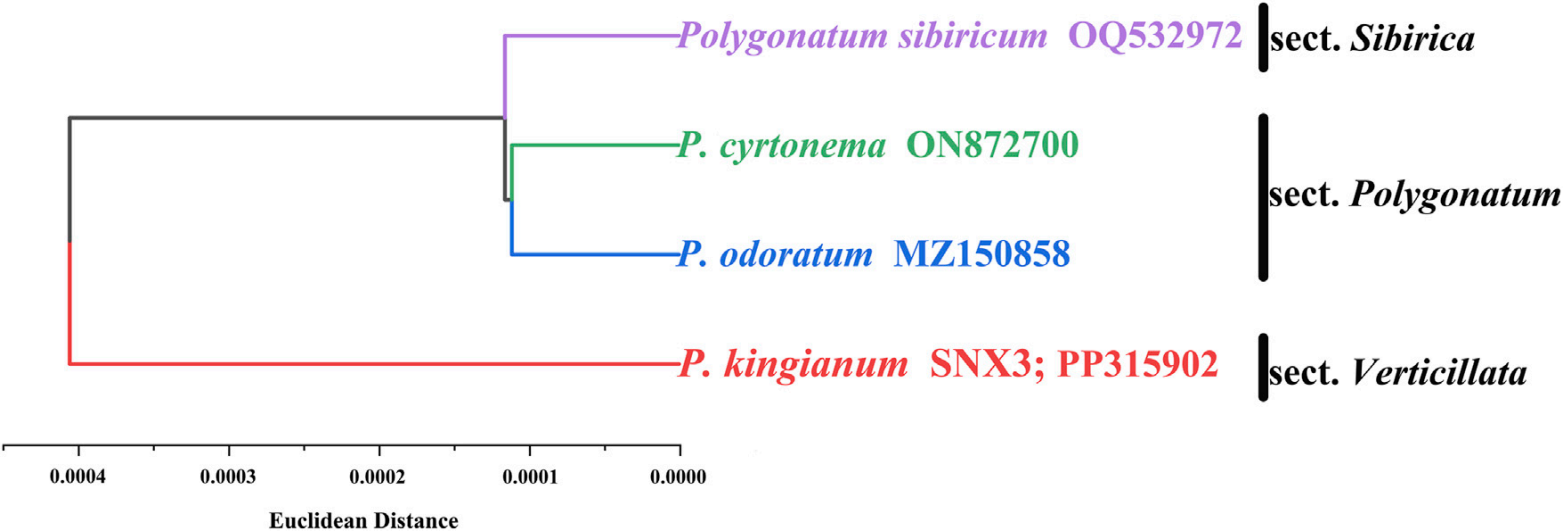
Neutrality plot analysis (GC12 vs. GC3) of cp genes in the medicinal *Polygonatum* species. R represents the Pearson’s correlation coefficient.

As shown in Supplementary Table S4, the value ranges of GC3 were 22.03%–37.36% (*P. cyrtonema*), 21.47%–37.12% (*P. kingianum*), 22.03%–37.24% (*P. odoratum*), 22.03%–37.42% (*P. sibiricum*), and the GC12 content of *P. cyrtonema*, *P. kingianum*, *P. odoratum* and *P. sibiricum* varied from 33.06% to 55.40%, 32.88%–55.76%, 33.09%–55.40%, 33.12%–55.40%, respectively. We constructed a neutrality plots (GC3 vs. GC12) for the cp genes in four medicinal *Polygonatum* species (Figure 5). The correlation coefficient (R) between GC12 and GC3 of these medicinal *Polygonatum* species were 0.0185 (*P. cyrtonema*), 0.0126 (*P. kingianum*), 0.0201 (*P. odoratum*), 0.0098 (*P. sibiricum*), respectively. The regression coefficients ranged from 0.1411 to 0.2057, indicating that the contribution of mutation pressure effect accounted for 14.11%–20.57%. The results of neutrality plot analysis suggest that the contribution of natural selection was more significant than that of mutation selection.

### 3.3 ENC-plot analysis

**FIGURE 6:**
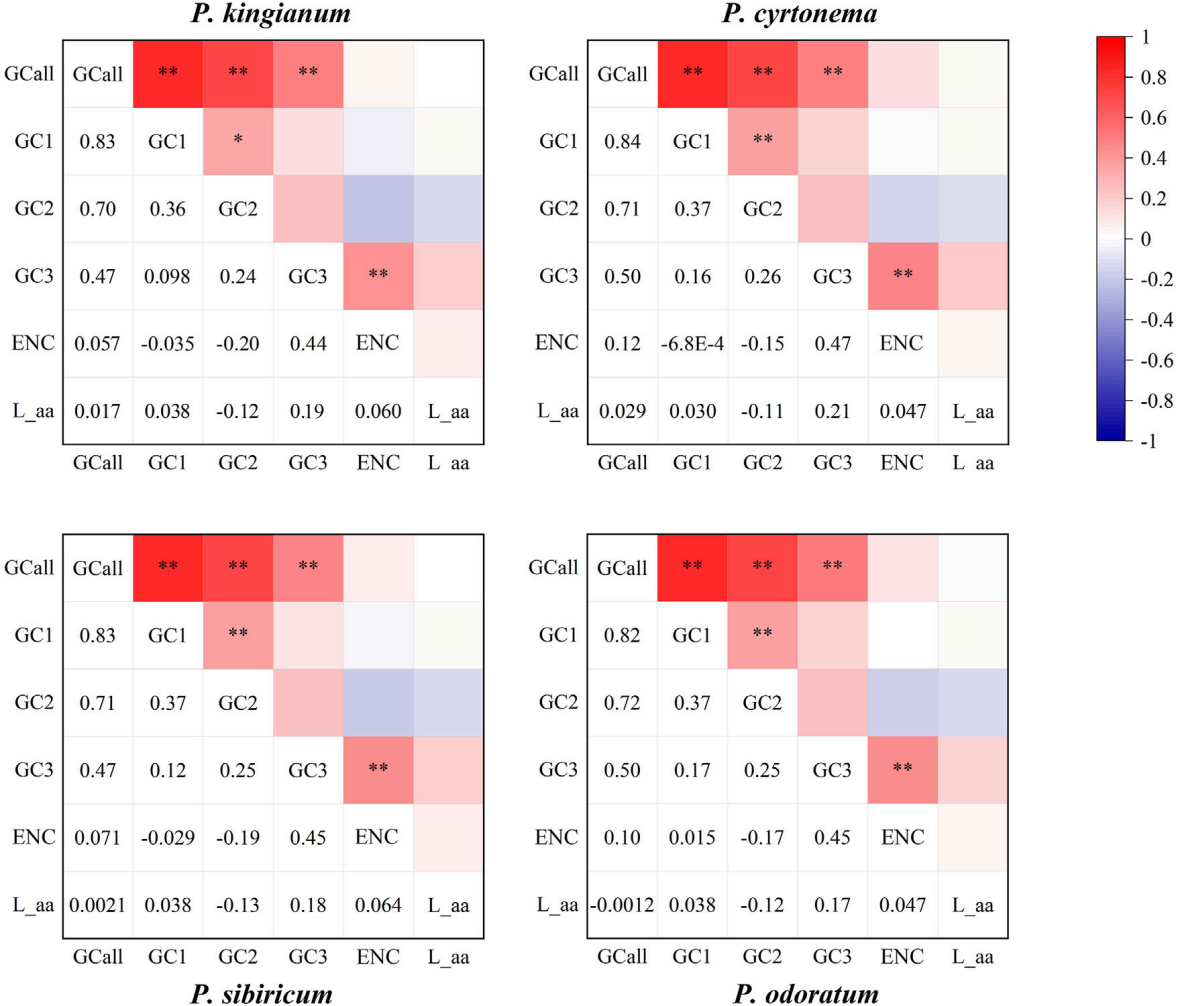
ENC-GC3s plot analysis of 51 tested cp genes in medicinal *Polygonatum* species. The yellow expected curve shows codon usage bias is only affected by mutation pressure.

The results of ENC-plot analysis of the four medicinal *Polygonatum* species were shown in Figure 6. All tested species had similar patterns in the ENC-plots. We found that most points were under the standard curve, and only a few were near or above the standard curve. It indicates that the CUB of most genes was mainly affected by selection pressure, while a few genes were affected by mutation pressure.

**FIGURE 7:**
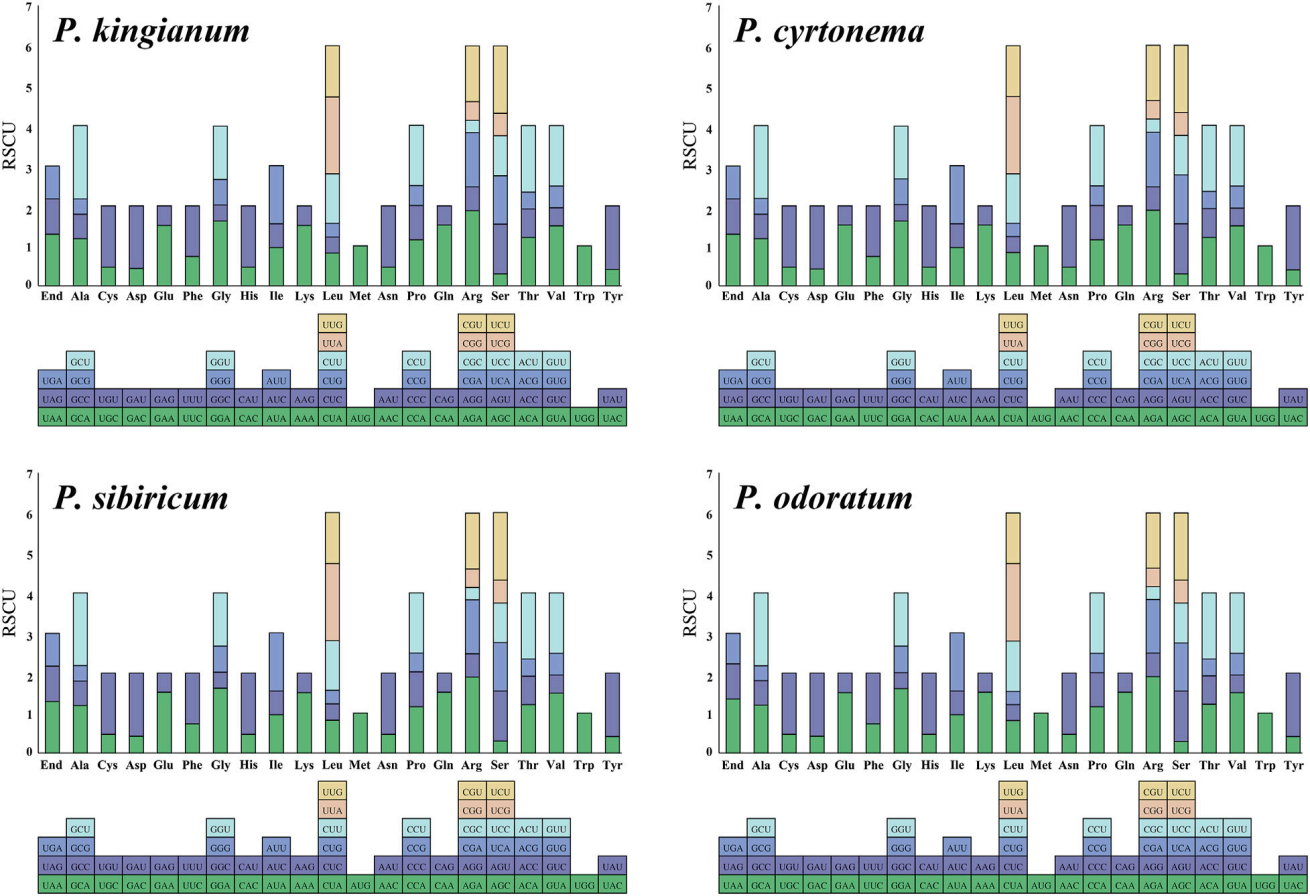
Cp genes number distribution of ENC frequency ratio.

The ENC ratio frequency was calculated to observe the variation range between the actual and expected values of ENC. Distribution of all sample’s ENC ratios was shown in Figure 7 and showed almost the same distribution. Majority of cp genes (30–31 genes) had ENC ratios between −0.05 and 0.05, meaning their CUB was primarily influenced by mutation pressure. In contrast, natural selection influenced the CUB of the remaining cp genes.

### 3.4 Parity rule 2 (PR2) plot analysis

**FIGURE 8:**
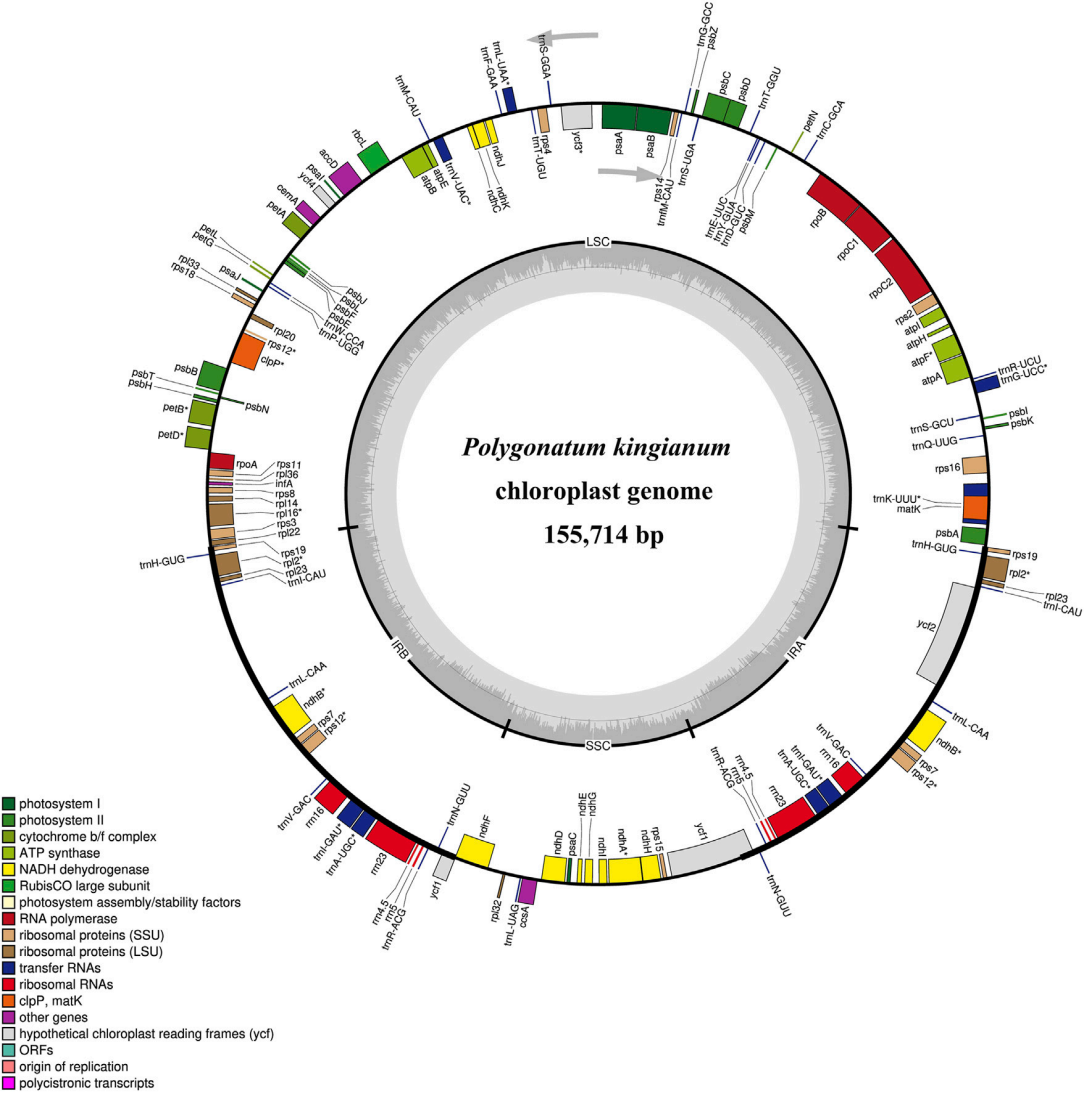
Parity Rule 2 (PR2) plot analysis of 51 cp genes in four medicinal *Polygonatum* species. The frequency of base A, T, G, and C at the third position of codons are denoted by A3s, T3s, G3s, C3s.

The PR2 plot analysis mainly analyzes the bias between the third base A, T, G, and C in chloroplast genes. The PR2-plots of the medicinal *Polygonatum* species were shown in Figure 8. As can be seen from the PR2-plot, the points of these medicinal *Polygonatum* species showed a similar distribution. Besides, the distribution of dots in the four quadrants was unbalanced, and most of the dots were distributed in the third and fourth quadrants. These results suggested a few differences in the same genes among different medicinal *Polygonum* species. And the third base position of the synonymous codon preferred to end in T/C. In summary, the formation of codon usage patterns was influenced by natural selection and mutation pressure.

### 3.5 Correspondence analysis (COA)

**FIGURE 9:**
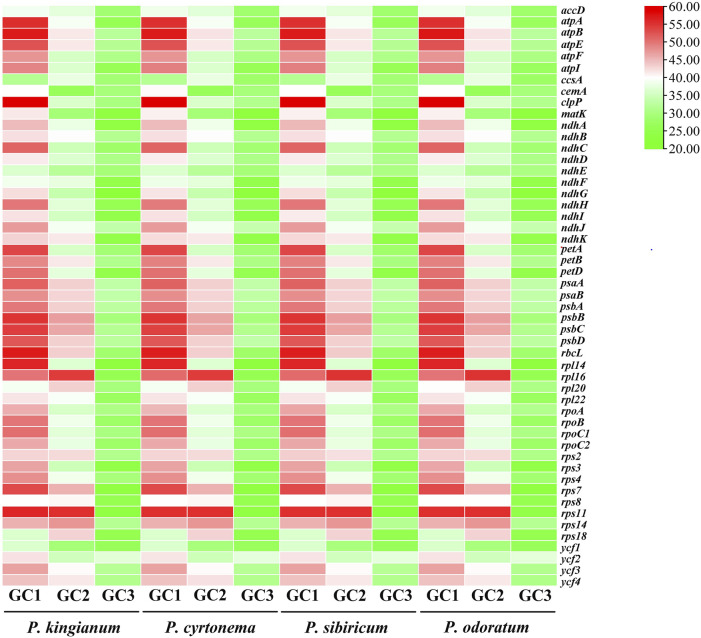
Spearman’s correlation analysis heatmap of different codon usage indicators of four medicinal *Polygonatum* species. The color of the color block changes from blue to red, indicating that the correlation index is rising. An asterisk indicates a significant association (*p* < 0.05); two asterisks indicate a extremely significant correlation (*p* < 0.01).

The correlation analysis of four medicinal *Polygonatum* species was shown in Figure 9. Correlation analysis results show that GCall was extremely significantly correlated with the GC1, GC2, and GC3 in all samples (*p* < 0.01). A extremely significant positive correlation was also observed between GC3 and ENC of all tested medicinal *Polygonatum* species (*p* < 0.01). In the three tested medicinal *Polygonatum* species (*P. odoratum, P. sibiricum, P. cyrtonema*), a extremely significant positive correlation was observed between GC1 and GC2 (*p* < 0.01). In *P. kingianum*, GC1 had a significant positive correlation with GC2 (*p* < 0.05). However, there was no correlation between GC3 and GC1 or GC2 in all medicinal *Polygonum* species. Furthermore, codon number (L_aa) was not significantly associated with all tested parameters. In all *Polygonatum* species tested, there was no significant correlation between ENC and GC1 or GC2.

### 3.6 Optimal codons of medicinal *Polygonatum* species chloroplast genes

We calculated the RSCU difference (△RSCU) per codon for high-expression and low-expression groups. According to the calculation results of △RSCU (Supplementary Table S6), 30 high-expression codons were screened in the each medicinal *Polygonatum* species. We detected 10 to 11 optimal codons based on high-frequency codons (RSCU>1) and high-expression codons (ΔRSCU>0.08). The results showed that there were differences in the number and types of the optimum codons of the medicinal *Polygonatum* species. The optimal codons of *P. cyrtonema* were AAU, ACA, AGA, CAU, CCA, CUU, GAU, GGA, UAU, UCA. The optimal codons of other medicinal *Polygonatum* species (*P. sibiricum*, *P. odoratum*, and *P. kingianum*) were AAU, ACA, CAA, CAU, CCA, CUU, GAU, GGA, UAU, UCA, UUU. Most of the optimal codons of medicinal *Polygonatum* species ended in A/U.

### 3.7 RSCU-based cluster analysis

**FIGURE 10:**
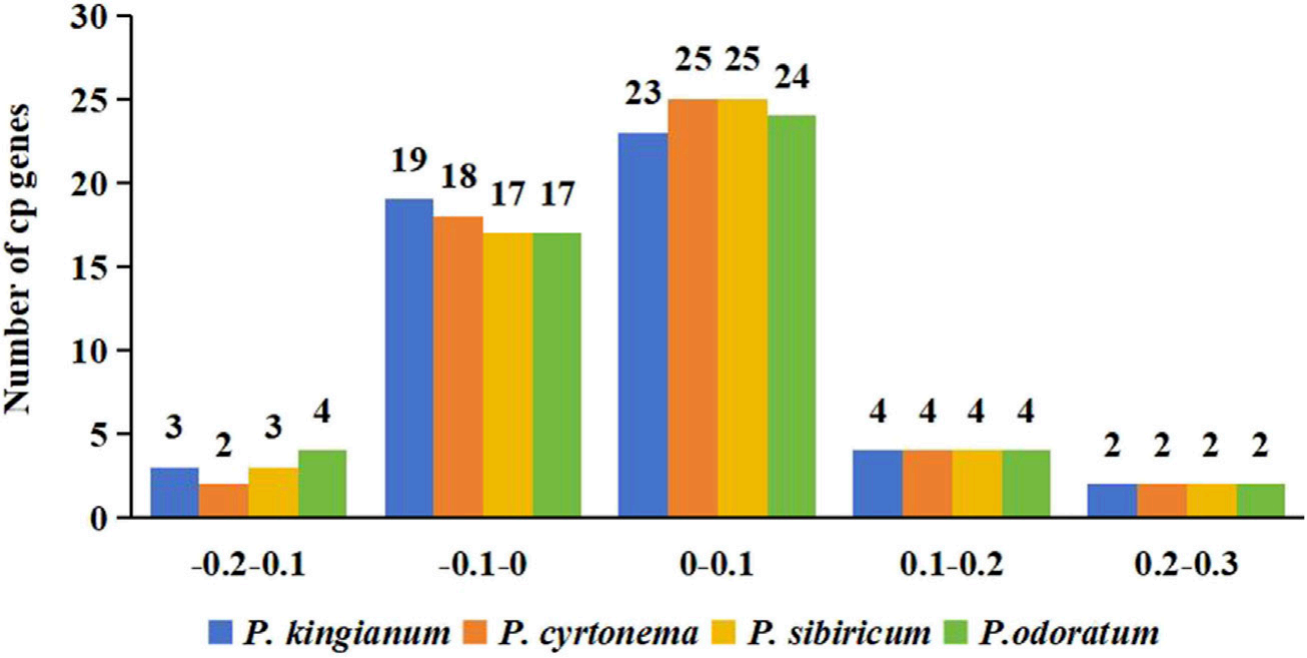
Hierarchical clustering analysis based on the RSCU of codons in chloroplast CDSs.

The codon-based hierarchical cluster tree is shown in Figure 10. *P. kingianum* (sect. *Verticillate*) was located at the outermost position of the tree, followed by the *P. sibiricum* (sect. *Verticillate*). Finally, the next node included the *P. cyrtonema* and *P. odoratum* from the sect. *Polygonatum*. Among the four medicinal species of the genus *Polygonatum*, the relationship between *P. cyrtonema* and *P. odoratum* was closer than *P. sibiricum*; the relationship with *P. kingianum* was weaker for *P. sibiricum*, *P. cyrtonema* and *P. odoratum*. The species relationship inferred based on RSCU is consistent with other studies built based on the sequences (Xia et al., 2022; Wang J et al., 2022).

## 4 Discussion

Genetic codon is the link between nucleic acid and protein, and plays an important role in the transmission of genetic information in organisms (Tang et al., 2021). Many studies have confirmed that codon bias varies in different species and genes which is considered to be an evolutionary behavior caused by adapting to their environment (Chaney et al., 2016; Zhang et al., 2019; Chakraborty et al., 2020; Shi et al., 2022). Previous studies have found that many specific factors affect codon preference, including gene length, gene expression level, tRNA abundance, mutation, selection, and so on, among which mutation and selection pressure are the main influencing factors (Wan et al., 2004; Meyer, 2021). Research on the codon bias characteristics and variations is of great significance for the understanding of molecular evolution and the exogenous expression of species (Wang Z et al., 2022; Chen et al., 2023).

In the present study, we systematically investigated the codon usage patterns of CDS in the medicinal *Polygonatum* chloroplast genomes, as well as the shaping factors influencing the codon usage bias patterns. Codon usage index analysis showed that the base composition shows different characteristics at the three codon positions. We found that the GCall and GC3 content of the tested chloroplast genomes was less than the AT content, which means these medicinal *Polygonatum* species exhibited a preference for the use of A or T nucleotides. This finding was consistent with a previous study that higher plants have a tendency to use codons that end in A or T (Campbell and Gowri, 1990). RSCU is an essential parameter in studying the codon usage bias of species (Sharp and Li, 1986). According to RSCU analysis, most high-frequency and highly-expressed codons also end with A/U, which further demonstrates the A/U preference trend of the third codon of medicinal *Polygonatum* species. ENC values analysis of chloroplast genomes revealed weak codon bias in these species. According to the ENC value, the codon preference of genes responsible for photosynthesis and self-replication is stronger than that of others. The reason for the difference of CUB among four kinds of genes is probably related to the adaptive adjustment of species to the environment. We also detected the correlation between GC content (GC1, GC2, GC3, GCall), ENC, and codon number (L_aa) from four medicinal *Polygonatum* spices, indicating the base composition influenced codon usage bias. Combined with analysis of the neutrality plot, ENC-plot, and PR2-plot, it revealed that both mutation pressure and natural selection had affected CUB in the chloroplast genomes of the four medicinal *Polygonatum* species, of which natural selection made great contributions in framing the CUB. Correlation analysis showed that GC3 has no correlation with GC1 or GC2, implying that the base composition in the third codon position significantly differed from those of the former two. Moreover, no correlation was observed between L_aa and ENC, indicating that gene sequence length has no effect on CUB. According to all the resulting data we obtained, the codon usage bias of chloroplast genomes was similar among the four *Polygonatum* species, indicating that natural selection and mutation pressure have similar effects between closely related species. This may be related to the conservation of cp genome evolution.

Codon optimization, which adjusts synonymous codons of foreign genes according to the codon usage pattern of the cp genome, to improve the efficiency and accuracy of related gene expression products (Tang et al., 2021). The 10-11 optimal codons were identified in medicinal *Polygonatum* spices chloroplast genomes. However, the optimal codon of *P. cyrtonema* was somewhat different from that of the remaining three species. The reason for the variations is due to the inconsistency between the defined high and low expression gene groups. The relationship between different medicinal *Polygonatum* species was constructed based on the RSCU values and found that the three species *P. sibiricum*, *P. cyrtonema*, and *P. odoratum* were closely related to each other, but distantly related to *P. kingianum*. We believe that the reason for this relationship may be due to the high geographical overlap among *P. sibiricum* (Northwestern, Northeastern, North, and South China), *P. cyrtonema* (Southwest, Central, East, North, and South China), and *P. odoratum* (Northwestern, Central, East, and North China), which is higher than that of *P. kingianum* (Southwest and South China) (Chen and Tamura, 2000). Therefore, it can provide a reference for inferring their genetic relationship according to the difference in RSCU values among different medicinal *Polygonatum* species. All the above results can provide a theoretical basis for subsequent exogenous gene improvement and genetic evolution in the chloroplast genome of medicinal *Polygonatum* species.

Codon bias is only one of many factors that affect gene expression. Furthermore, CUB itself is influenced by a variety of factors. Hence, although CUB has important applications in enhancing heterologous gene expression, it has not been well studied in most medicinal plants. To date, the RSCU-based cluster analysis has been applied to explore the relationship of plants. However, some studies have shown that RSCU clustering results were inconsistent with CDS phylogenetic tree results (Niu et al., 2021; Zhang et al., 2024). Therefore, more *Polygonum* species are needed to examine its reliability in future studies.

## 5 Conclusion

This was the first report to systematically compare codon usage characteristics and patterns in the chloroplast genomes of medicinal *Polygonatum*. In the present study, 204 PCGs from chloroplast genomes of four *Polygonatum* species were screened and calculated to analyze the CUB. The results of the present study exhibited similar weak codon usage preferences prevalent in chloroplast genomes of these *Polygonatum* species. We discussed the formation of codon usage patterns of medicinal *Polygonatum* species and confirmed that natural selection was the determining factor affecting codon preference. Besides, the mutation factor could not be ignored. Based on the analysis results of CUB, we identified 10-11 optimal codons in these chloroplast genomes of four *Polygonatum* species, and most of them ending with A or U. Our finding indicated that the phylogenetic results based on RSCU cluster analysis not only can be used for understanding the evolutionary relationships of different medicinal *Polygonatum* species but also can be an important supplement to the phylogenetic results based on sequences. In summary, this study provides an important theoretical reference about the codon usage tendency and variation, codon optimization of exogenous genes, as well as evolutionary analysis in these important medicinal *Polygonatum* species.

## Data Availability

The datasets presented in this study can be found in online repositories. The names of the repository/repositories and accession number(s) can be found in the article/supplementary material.
